# Thyroid Autoimmunity and Behçet's Disease: Is There a Significant Association?

**DOI:** 10.1155/2013/956837

**Published:** 2013-02-07

**Authors:** Filiz Cebeci, Nahide Onsun, Ayşe Pekdemir, Ahmet R. Uras, Kadir Kayataş

**Affiliations:** ^1^Department of Dermatology, Haydarpaşa Numune Training and Research Hospital, 34668 İstanbul, Turkey; ^2^Department of Dermatology, Faculty of Medicine, Bezmialem Vakıf University, İstanbul, Turkey; ^3^Biochemistry Laboratory, Haydarpaşa Numune Training and Research Hospital, 34668 İstanbul, Turkey; ^4^Department of General Medicine, Haydarpaşa Numune Training and Research Hospital, 34668 İstanbul, Turkey

## Abstract

*Background*. Behcet's disease (BD) could be regarded as an autoimmune disease in many aspects. Autoimmune thyroid disease (ATD) is frequently accompanied by other various autoimmune diseases. Nevertheless, there is not still enough data showing the association between BD and ATD. In addition, no controlled study is present in the PubMed, which evaluates thyroidal autoimmunity using antithyroid peroxidase antibody in a large series of patients with BD. *Methods*. We aimed to investigate the frequency of ATD in patients with BD. The study included 124 patients with BD and 99 age- and sex-matched healthy volunteers. *Results*. Autoimmune thyroiditis was noted in 21 cases (16.9%) with BD. In the control group, 22 cases (22.22%) were diagnosed as autoimmune thyroiditis. There was no difference between the groups in respect to thyroid autoantibodies (*P* > 0.05). There were no statistically significant differences between baseline TSH levels of the BD patients and of the controls (*P* > 0.05). Statistically, the mean serum free T4 levels of the patients with BD were higher than those of the controls (*P* < 0.001). *Conclusions*. No association could be found between BD and ATD. Therefore, it is not of significance to investigate thyroid autoimmunity in BD.

## 1. Introduction

Behçet's disease (BD) is a chronic, multisystemic vasculitic disease characterized by recurrent orogenital ulcerations, uveitis, and skin lesions. However, the disease may affect any organ system in the body during its clinical course [1]. Vasculopathy of both arteries and veins of all sizes is a common complications associated with BD. The main histopathological feature of the disease is vasculitis [2, 3]. Therefore, the organs, including the thyroid gland with rich vascular supply, is likely to be affected easily by vasculitic events. The ethiopathogenesis of BD remains to be understood, although the role of autoimmunity in BD has been widely discussed [4–6]. 

 It is well known that the thyroid gland is a frequent target of autoimmune activation. The condition develops as an autoimmune response against thyroid autoantigens. Anti-TPO and, to a lesser extent, anti-TG are sensitive tests for assessing thyroidal autoimmunity. In addition, autoimmune thyroid disease (ATD) is often associated with other autoimmune conditions [7, 8]. The association between rheumatologic disorders such as systemic lupus erythematosus, Sjögren's syndrome, and thyroid disorders has long been known [9, 10]. However, in literature, there is very little data on the thyroid autoimmunity in BD. Given that the thyroid may be affected by both vasculitis and by the autoimmune background of Behçet's disease, we aimed to investigate the relationship between BD and ATD. 

## 2. Patients and Methods 

The study was designed as a prospective and approved by the local ethical committee. All patients were informed about the study protocol and gave their written consents. From February 2009 to August 2011, 124 consecutive patients with Behçet's disease (mean age 38, 481 ± 0, 52 years; range from 15 to 69 years; 38 females; 86 males) and 99 healthy volunteer controls (mean age 37, 691 ± 0, 86 years; range from 19 to 71 years; 42 females; 57 males) were enrolled in this study. The study group comprised patients who attended the dermatology outpatient clinic of Vakıf Gureba Teaching Hospital. All BD patients fulfilled the International Study Group criteria for the diagnosis of BD [11]. Neither patients nor controls had taken any drugs such as systemic corticosteroid or dopamine during the previous 6 months. The control group was composed of subjects who were age-and sex-matched patients with Behçet's disease.

 In both the patient and control groups, thyroid stimulating hormone (TSH), free thyroxine (FT_4_), antithyroperoxidase (TPO), antithyroglobulin (TG) were measured. TSH and FT_4_ were measured by chemiluminescent immunometric assay (DPC, USA). TPO and TG antibodies were measured by chemiluminescent immunometric assay (DPC, USA). The normal reference range for anti-TPO was 0–115 IU/mL and for anti-TG was 0–34 IU/mL. Diagnosis of ATD was made according to thyroid autoantibody levels (anti-TPO and/or anti-TG) [12]. The normal reference range for TSH was 0.4–4.0 *μ*g/dL and for FT_4_ was 0.8–1.9 ng/dL. The diagnosis of hypothyroidism was made by clinical and laboratory criteria of FT_4_ < 0.8 ng/dL, TSH > 4.0 *μ*g/dL. The patients were classified as having subclinical hypothyroidism if they were clinically euthyroid with normal FT_4_ levels but had significantly elevated TSH levels. The diagnosis of hyperthyroidism was based on the detection of low-serum TSH values (<0.4 *μ*g/dL) and elevated levels of FT_4_ (>1.9 ng/dL) in serum. The study group were evaluated by an internist dealing with thyroid diseases. Ultrasonography examination was performed on patients who were found to have enlarged thyroid gland and increased thyroid function tests and thyroid antibody in physical examination. 

 NCSS 2007 (Number Cruncher Statistical System) and PASS 2008 statistical software programme, Student's *t*-test, Chi-square, and Mann-Whitney *U* tests were used for the statistical analysis of the data. *P* < 0.05 was taken as statistically significant.

## 3. Results

Because the patients were age- and sex-matched with the control group, there was no statistical difference between the controls and the patients with respect to the age and sex. Anti-TG antibody was higher than the normal antibody titers in 14 patients, anti-TPO antibody in 4 (3.77%), and both TG and TPO antibodies in 3 (3.85%). In patients with BD anti-TG titers ranged from 100 to 990 IU/mL and anti-TPO antibody titers from 279 to 340 IU/mL. Autoimmune thyroiditis was noted in 21 cases (16.9%) with BD. In the control group, 22 cases (22.22%) were diagnosed with autoimmune thyroiditis. There was no statistically significant difference with regard to both TPO and TG antibodies between patients with BD and the control group (*P* < 0.05).  Table 1 shows the frequency of thyroid antibodies in the study group.

 Among the patients with autoimmune thyroiditis, 17 (80.95%) were euthyroid, 2 (9.52%) cases were subclinically hypothyroid, one (4.76%) was hypothyroid autoimmune thyroiditis (Basedow's disease), one (4.76%) was hyperthyroid, and autoimmune thyroiditis was diagnosed (Graves' disease). Three patients with autoimmune thyroiditis (one euthyroid, one subclinically hypothyroid, one hypothyroid autoimmune thyroiditis) were found to have goiter on USG. Four of 22 controls with autoimmune thyroiditis had thyroid dysfunction (three subclinically hypothyroid, one hypothyroid autoimmune thyroiditis) and the other eighteen cases were euthyroid. Compared to the controls, FT_4_ values of patients with BD were determined to be significantly lower. However, there was no finding suggesting the clinical signs of primary or secondary hypothyroidism in our patients. There was no statistically significant difference with regard to TSH between patients with BD and the control group.  Table 2 shows thyroid hormone levels and autoimmune thyroiditis status in the patient and control groups. 

## 4. Discussion

Behçet's disease can be regarded as an autoimmune disease with reference to its many features including spontaneous remissions and relapses as well as its response to classical immunosuppressants like azathioprine and cyclophosphamide similar to various autoimmune diseases [4, 13]. As autoimmunity is blamed in the etiology of BD, the possibility of increased incidence of autoimmune thyroiditis should be considered. The occurrence of different autoimmune diseases simultaneously in the same patient is more frequent than is usually believed. The existence of ATD among patients with autoimmune diseases such as vitiligo, chronic urticaria, and autoimmune rheumatic diseases has been well recognized [14–17]. The simultaneous occurrence of different autoantibodies in different diseases does not seem to be coincidental but to result from similar immunopathogenic mechanisms involved in these disorders, which are still not completely understood [4, 18].

 In contrast to other autoimmune rheumatic diseases, our study showed that the frequency of autoimmune thyroiditis was not higher in patients with BD. There was only one controlled study investigating the relationship between BD and thyroid autoimmunity in Pubmed. In this above-mentioned study with a small number of patients by Aksu et al., there was no significant association between BD and incidence of thyroid autoantibodies just like in our results except for decreased level of free T_4_ [19]. In their study anti-TG autoantibody as well as thyroid microsomal were used, which are less sensitive and specific than antithyroperoxidase antibodies. 

 Our study suggested that a larger series of patients did not change the result using anti-TPO, which is more sensitive and specific in determining the autoimmune thyroiditis, and this supported the data raising questions about the place of BD in autoimmune diseases. According to these data, Behçet's disease does not have the classical clinical features of autoimmunity such as anti-nuclear antibody positivity, female dominance, and any association with other autoimmune diseases such as Sjögren's syndrome, vitiligo. Another features distinguishing BD from autoimmune conditions are the lack or paucity of serositis, glomerulonephritis, Raynaud's phenomenon, the lack of organ-specific, nonspecific autoantibodies, B-cell hyperfunction, T-cell hypofunction, the lack of HLA alleles associated with autoimmune diseases, and a distinct geographic distribution [4, 13, 20].

 On the other hand, the association between BD and other autoimmune diseases is not well documented in the literature. For example, case reports have been published on the concomitant occurrence of BD and autoimmune diseases such as Sjögren's syndrome diabetes insipidus [21, 22]. To our knowledge, there is only one study in the literature investigating the association between vitiligo and BD [19]. This study did not find any relation between vitiligo and BD, either. Cho et al. examined the presence of autoimmune disease in many BD patients and they reported 11 cases with various autoimmune diseases among their 473 patients with BD [23]. Of 11 patients, four had autoimmune thyroiditis. However, this study did not include a control group and did not use anti-TPO autoantibodies, which might be a defect for the reliability of the study. 

 We assume that BD does not seem to share a common autoimmune mechanism for autoimmune thyroiditis. However, only free T_4_ levels were significantly lower in patients with Behçet's disease in our study, which suggests that thyroid parenchyma might be affected by vasculitic process. On the other hand, morphological changes are likely to occur the thyroid gland due to vasculitis, even if in the absence of functional changes. As a result, we think that thyroid parenchyma should be further investigated, whereas it is not of value to investigate thyroid autoimmunity in Behçet's disease. The lack of an association between autoimmune thyroiditis and BD constitutes further evidence that conventional autoimmune mechanism may not be effective in BD.

## Figures and Tables

**Table 1:** Frequency of thyroid autoantibodies in the study group.

	Anti-TG	Anti-TPO	Anti-TG and anti-TPO	Total
Patients	14 (18.42%)	4 (3.77%)	3 (3.85%)	21 (16.9%)
Controls	15 (26.79%)	4 (4.88%)	3 (5.46%)	22 (22.22%)

**Table 2:** Thyroid hormone levels and autoimmune thyroiditis status in the patient and control groups.

	Euthyroid (*n*)	Subclinicd hypothyroid (*n*)	Hypothyroid (*n*)	Hyperthyroid (*n*)	FT_4_ (mean ± SD)	TSH (mean ± SD)
Patients *n* = 124	17 (80.95%)	2 (9.52%)	1 (4.76%)	1 (4.46%)	1.21 ± 0.19	1.67 ± 1.08
Controls *n* = 99	18 (81.82%)	2 (9.10%)	1 (4.54%)	1 (4.54%)	1.11 ± 0.19	1.83 ± 0.98
*P *					0.000*	0.120

Student's *t-*test was used. *P < 0.001.

## References

[1] Barnes CG, Yazici H (1999). Behçet's syndrome. *Rheumatology*.

[2] Lie JT (1992). Vascular involvement in Behcet’s disease: arterial and venous and vessels of all sizes. *Journal of Rheumatology*.

[3] Koç Y, Güllü I, Akpek G (1992). Vascular involvement in Behçet’s disease. *The Journal of Rheumatology*.

[4] Yazici H (1997). The place of Behçet’s syndrome among the autoimmune diseases. *International Reviews of Immunology*.

[5] Gül A (2005). Behçet’s disease as an autoinflammatory disorder. *Current Drug Targets: Inflammation and Allergy*.

[6] Yazici H, Fresko I (2005). Behçet’s disease and other autoinflammatory conditions: what’s in a name?. *Clinical and Experimental Rheumatology*.

[7] Weetman AP (2009). The genetics of autoimmune thyroid disease. *Hormone and Metabolic Research*.

[8] Weetman AP, McGregor AM (1994). Autoimmune thyroid disease: further developments in our understanding. *Endocrine Reviews*.

[9] Pyne D, Isenberg DA (2002). Autoimmune thyroid disease in systemic lupus erythematosus. *Annals of the Rheumatic Diseases*.

[10] Pérez B, Kraus A, López G (1995). Autoimmune thyroid disease in primary Sjögren's syndrome. *American Journal of Medical Genetics*.

[11] International Study Group for Behcet's Disease (1990). Criteria for diagnosis of Behçet's disease. *The Lancet*.

[12] Saravanan P, Dayan CM (2001). Thyroid autoantibodies. *Endocrinology and Metabolism Clinics of North America*.

[13] Direskeneli H (2006). Autoimmunity vs autoinflammation in Behcet’s disease: do we oversimplify a complex disorder?. *Rheumatology*.

[14] Amerio P, Di Rollo D, Carbone A (2010). Polyglandular autoimmune diseases in a dermatological clinical setting: vitiligo-associated autoimmune diseases. *European Journal of Dermatology*.

[15] Cebeci F, Tanrıkut A, Topcu E, Onsun N, Kurtulmus N, Uras AR (2006). Asssociation between chronic urticaria and thyroid autoimmunity. *European Journal of Dermatology*.

[16] Punzi L, Betterle C (2004). Chronic autoimmune thyroiditis and rheumatic manifestations. *Joint Bone Spine*.

[17] Soy M, Guldiken S, Arikan E, Altun BU, Tugrul A (2007). Frequency of rheumatic diseases in patients with autoimmune thyroid disease. *Rheumatology International*.

[18] Nisihara RM, de Bem RS, Hausberger R (2003). Prevalence of autoantibodies in patients with endemic pemphigus foliaceus (fogo selvagem). *Archives of Dermatological Research*.

[19] Aksu K, Oksel F, Keser G (1999). Thyroid functions in Behcet’s disease. *Clinical Endocrinology*.

[20] Oran M, Hatemi G, Tasli L (2008). Behçet's syndrome is not associated with vitiligo. *Clinical Experimental Rheumatolgy*.

[21] Harifi G, Habib Allah M, Younsi R, El Hassani S (2009). Primary Gougerot-Sjögren syndrome and Behçet disease: association or coincidence?. *La Presse Médicale*.

[22] Jin-No M, Fuji T, Jin-No Y, Kamiya Y, Okada M, Kawaguchi M (1999). Central diabetes insipidus with Behçet’s disease. *Internal Medicine*.

[23] Cho SB, Chun EY, Bang D, Lee KH, Lee ES, Lee S (2005). Coexistence of Behçet's disease and autoimmune disease: clinical features of 11 cases. *The Journal of Dermatology*.

